# Characterizing Complex Networks Using Entropy-Degree Diagrams: Unveiling Changes in Functional Brain Connectivity Induced by Ayahuasca

**DOI:** 10.3390/e21020128

**Published:** 2019-01-30

**Authors:** Aline Viol, Fernanda Palhano-Fontes, Heloisa Onias, Draulio B. de Araujo, Philipp Hövel, Gandhi M. Viswanathan

**Affiliations:** 1 Institute of Theoretical Physics, Technische Universität Berlin, Hardenbergstraße 36, 10623 Berlin, Germany; 2 Bernstein Center for Computational Neuroscience Berlin, Humboldt-Universität zu Berlin, Philippstraße 13, 10115 Berlin, Germany; 3 Brain Institute, Universidade Federal do Rio Grande do Norte, Natal–RN 59078-970, Brazil; 4 School of Mathematical Sciences, University College Cork, Western Road, T12 XF62 Cork, Ireland; 5 Department of Physics, Universidade Federal do Rio Grande do Norte, Natal 59078-970, Brazil; 6 National Institute of Science and Technology of Complex Systems Universidade Federal do Rio Grande do Norte, Natal 59078-970, Brazil

**Keywords:** entropy, functional brain networks, psychedelic state, Ayahuasca, complex networks

## Abstract

With the aim of further advancing the understanding of the human brain’s functional connectivity, we propose a network metric which we term the *geodesic entropy*. This metric quantifies the Shannon entropy of the distance distribution to a specific node from all other nodes. It allows to characterize the influence exerted on a specific node considering statistics of the overall network structure. The measurement and characterization of this structural information has the potential to greatly improve our understanding of sustained activity and other emergent behaviors in networks. We apply this method to study how the psychedelic infusion Ayahuasca affects the functional connectivity of the human brain in resting state. We show that the geodesic entropy is able to differentiate functional networks of the human brain associated with two different states of consciousness in the awaking resting state: (i) the ordinary state and (ii) a state altered by ingestion of the Ayahuasca. The functional brain networks from subjects in the altered state have, on average, a larger geodesic entropy compared to the ordinary state. Finally, we discuss why the geodesic entropy may bring even further valuable insights into the study of the human brain and other empirical networks.

## 1. Introduction

In the last few decades, new scientific fields have taken advantages of complex network approaches. This interest emerged, in part, by virtue of technological advances that generate new datasets in computational, social, biological, and others sciences [1,2,3]. Examples include modern brain mapping techniques, such as functional magnetic resonance imaging (fMRI), that have provided previously inaccessible information about interaction patterns in the human brain [4]. The theory of complex networks has proven to be a crucial tool to understand the interactions and dynamics in large systems.

Attempts to characterize new datasets bring up the challenge of extracting relevant features regarding the network’s structure. One of the main concerns is to identify the role of each node in the network and how the nodes cooperate to give rise to emergent behaviors. The majority of measurements that have been proposed in the last few decades allow the ranking of nodes’ importance by the number of connections, centrality, etc. [5,6,7].

Instead of ranking a node’s relative importance, we ask how the nodes contribute locally to the global connectivity of the network, with the aim of better understanding the individual role played by each node in the network. We wish to characterize this role by quantifying the structural information of the diversity of influences among nodes.

**Figure 1:**
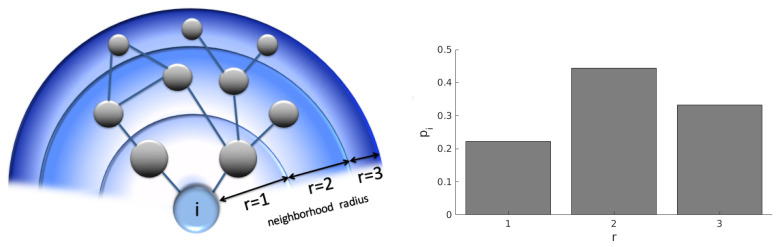
Schematic representation of neighborhood radius *r* and its probability distribution. The left panel shows three neighborhood radii for the node *i*. The nodes within neighborhoods r=1, r=2, r=3 are, respectively, 1, 2 and 3 links distant from the node *i*. On the right is the probability distribution of the geodesic distances from the node *i* for this network.

The nodes in a network interact with their neighbors and, indirectly, with the neighbors of neighbors; and also with more distant nodes with even greater “neighborhood radius” (Figure 1).

We aim to quantify the diversity of radii or distance of influences on a given node exercised by all other nodes in the network. For each node, we calculate the Shannon entropy functional [8] of the probability distribution of the geodesic distances, that is, the shortest path lengths between each node and all other nodes. We call this measurement *geodesic entropy*. Nodes with a great diversity of influences (i.e., with high geodesic entropy) may play an important role in, for example, to guaranteeing specialization of functional patterns. Besides, nodes with a low diversity of influences may indicate constraints relevant to network robustness. The statistical details of the distribution of distances, quantified by the Shannon entropy, may be a key to understanding how emergent behaviors arise.

We illustrate and apply our method to real network data. We use the geodesic entropy to analyze human brain functional networks under the influence of the psychedelic brew Ayahuasca.

Ayahuasca is a sacred brew from Amazonian indigenous culture made by decoction of two plants from Amazonian flora—the leaves of the bush Psychotria Viridis, that contains N, N-Dimethyltryptamine (DMT), and the vine Banisteriopsis caapi, that contains monoamine oxidase inhibitors MAOi [9]. The DMT is a serotonergic psychedelic similar to LSD and mescaline [10,11] but fast metabolized by the human body. The MAOi’s act slowing down this degradation, allowing the DMT to cross the blood-brain barrier and enabling hours of psychedelic experience [9]. For more information about Ayahuasca we refer [12,13,14,15,16].

Ayahuasca ingestion may cause deep changes in the cognition and perceptions, promoting substantial alterations in the sense of the reality and the self [16,17]. According to the neural correlate hypothesis, we expect to find features of functional brain networks that can be correlated to this specific consciousness state. We evaluate the networks extracted from fMRI data acquired from the same group of subjects in two sections: before and 40 minutes after Ayahuasca intake. The geodesic entropy is able to identify a specific behavior for networks related to the psychedelic state of consciousness: the nodes of functional brain networks under Ayahuasca effects tend to have a greater geodesic entropy than the ordinary condition.

Our most important contribution here, regarding the method, is the geodesic entropy. The mean (or first moment) geodesic distance is a standard and fundamental quantity of interest in the study of complex networks. In addition to the first moment of the geodesic distance, however, there is much insight to be gained from studying other functionals (e.g., higher moments) of its distribution. In this context, one of the most important functionals of a probability distribution is the Shannon entropy. This crucial point motivates our definition of the geodesic entropy. Moreover, we define this entropy for each node. For a network of *N* nodes, the geodesic entropy of all nodes is an *N*-dimensional vector. Our second key contribution regarding methodological novelty is the entropy-degree diagram, which is a method for projecting the *N*-dimensional geodesic distance vectors onto a 2-dimensional plot, with node degree being the second dimension. The entropy-degree diagram is a visual representation of both the connectivity (i.e., degree) as well as the entropy for each node: 2 *N*-dimensional vectors are projected as *N* points in 2 dimensions.

This paper is organized as follow: In Section 2 we define the metric geodesic entropy and introduce the entropy-degree diagram. In Section 3 we present the results regarding the use of geodesic entropy to comparing functional brain networks of individual before and after Ayahuasca intake. We present our conclusion and discussion in Section 4. In Section 5, we give information about the fMRI data and its processing to generate the functional networks.

## 2. Geodesic Entropy

A complex network is a schematic representation of the relations (links) between elements (nodes) of a system with a nontrivial topology of interactions [1,18,19]. Consider a non-weighted undirected network G(V,E), where *V* is a set with *N* nodes and *E* is the set of links. It is represented numerically by a N×N adjacency matrix $A_{i,j}$: If a pair of nodes *i* and *j* are connected, the matrix element is $A_{i,j}=1$ and $A_{i,j}=0$ otherwise. The nodes are connected if the elements that they represent share some kind of information, or have potential to share, have some kind of link, correlation or mutual influences. The number of links that have each node is termed degree. The statistics of the degrees in a network is quantified by the degree distribution, a histogram of degrees considering the whole network [18].

Nodes directly connected are called nearest first neighbors. A node can also influence and be influenced by the neighbors of its neighbors, called second nearest neighbors. Considering a connected network, the influences may be extended to all neighborhood radius. Our goal is to quantify the amount of information involved in the diversity of influence extending over the network. For this purpose, we calculate the Shannon entropy [8] considering the statistics of distances between a node and all their neighborhood radius.

Distances in network theory are related to the path lengths. By definition, a path length $\Gamma_{i,j}$ is the number of consecutive links between the pair of nodes *i* and *j*, following a specific trail. The shortest path length ($D\left(i,j\right)=\min(\left\{\Gamma_{i,j}\right\})$) defines geodesic distance between two nodes [20]. The geodesic distance has been used in several network characterizations such as small-world networks [21].

By looking at the distribution of geodesic distances for a given node, we can know how far or near that particular node is to all other nodes and thereby obtain a qualitative understanding of how that node fits into the network overall.

We define $p_{i}\left(r\right)$ as a probability mass function of find a node in the neighborhood ratio *r* of the node *i*, that is, the probability that, in a random choice, one selects a node *j* from the set of the remaining nodes $\bar{V}=\left\{j\right|j\in V\setminus \left\{i\right\}\}$ with the geodesic distance D(i,j)=r. This probability is defined as:

(1) $$p_{i}\left(r\right)=\frac{1}{\left(N-1\right)}\sum_{j\in \bar{V}}\delta_{D(i,j),r}\;,$$

where the neighborhood radius *r* assumes values according to the interval $1\leq r\leq r_{\max}$ with $r_{\max}=\max_{j\in \bar{V}}\left(D\left(i,j\right)\right)$. See an illustration in Figure 1.

The distribution $p_{i}\left(r\right)$ contains information about the connectivity across multiple links of a network. For illustration, consider hypercubic lattices of dimension *D* with links only between neighboring nodes. The distribution $p_{i}\left(r\right)$ scales according to $p_{i}\left(r\right)∼r^{D-1}$, because nodes a fixed distance *r* away lie on the (hyper)surface of constant distance to the node *i*, where in *D* dimensions, this surface has dimension D−1. Hence, it makes sense that the characterization of the distribution $p_{i}\left(r\right)$ has the potential to provide insights into network connectivity.

The geodesic entropy is given by:

(2) $$s_{i}\left[p_{i}\right]=-\sum_{r=1}^{r_{\max}}p_{i}\left(r\right)\log p_{i}\left(r\right)\;.$$

The value of $s_{i}$ does not depend on the network size for larger networks ($N\gg r_{\max})$. The characteristic geodesic entropy of a network is defined by:

(3) $$S=\frac{1}{N}\sum_{i=1}^{N}s_{i}\;.$$

Distinct from the entropy of the degree distribution that quantifies the constraints imposed by the network degree distribution [22], the geodesic entropy quantifies the information due to the intrinsic configuration of network structure. Networks with different structures can share the same degree distribution, that is, they can be degenerate in the entropy of the degree distribution. The characteristic geodesic entropy can lift the degeneracy of those networks. In this sense, the geodesic entropy is a more appropriate metric for characterizing the interdependence of influences in the network. For a comparison of the geodesic entropy with other similar metrics, we refer the reader to the discussion section.

### Entropy-Degree Diagram

**Figure 2:**
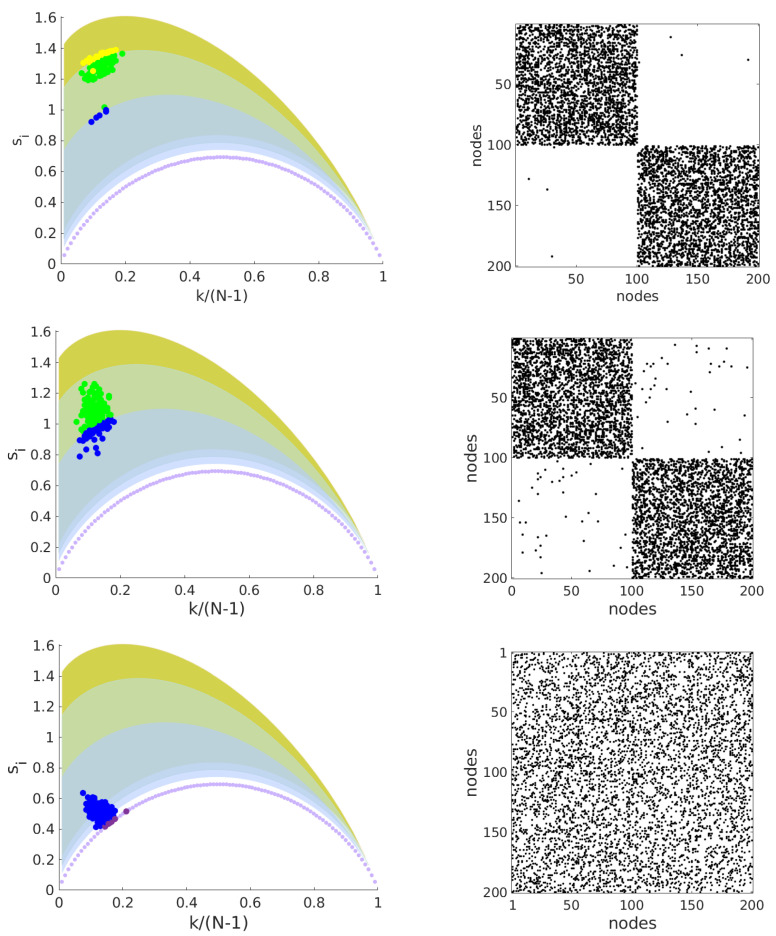
Illustration of entropy-degree diagram for three different artificially generated networks with the same number of nodes, links, same degree distribution (〈k〉=25), but different structural configurations. On the right side of each entropy-degree diagram is the adjacency matrix of the corresponding network. The characteristic geodesic entropy are S=1.26nat, S=0.98nat, S=0.52nat respectively from panels up to down. The colors purple, blue, green and yellow are defined according to the maximum neighborhood radius $r_{max}=2,3,4$ and 5. The shaded regions delimit the space of possibilities for each value of the maximum neighborhood. The minimum entropies possible are delimited by the purple curve and depends on the node degree.

**Figure 3:**
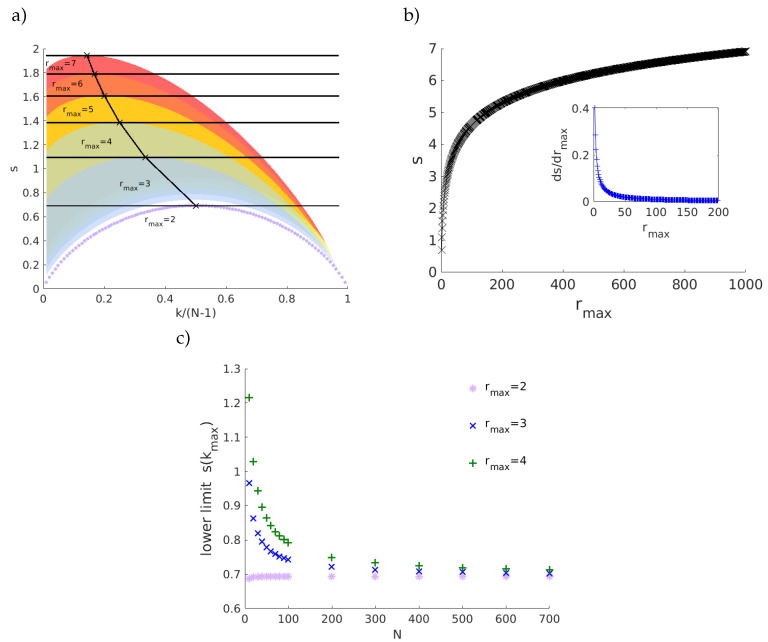
Universal relations for geodesic entropy. In the entropy-degree diagram in panel (**a**), the colored regions delimit the regions of nodes with maximum neighborhood values ($r_{\max}$) indicated by the labels. The maximum value of each region is in $s_{i}\approx \ln\left(r_{\max}\right)$, that correspond to $k\approx 1/r_{\max}$. Panel (**b**) shows the variance in the maximum entropy due the increase of neighborhood radius $r_{\max}$. Its increases are inversely proportional to $r_{\max}$ ($\Delta s_{\max}\approx r_{\max}^{-1}\Delta r_{\max}$). Note that for small values of $r_{\max}$, its increasing will result in an increase in contribution to the entropy. Nevertheless, for large $r_{\max}$ the contribution does not change significantly. Note that none of these values depend on network size. The finite size effect appears in the lower limit. For large networks, all regions will be delimited by the first curve ($r_{\max}=2$). The lower limit will depend on the network size for finite networks. The plot (**c**) shows the influence of network size in the lower limits.

Next, we introduce the entropy-degree diagram, a visualization tool to depict each node along two axes: (i) geodesic entropy and (ii) node degree. Entropy-degree diagram is built plotting the geodesic entropy ($s_{i}$) *versus* the nodal degree *k* normalized by the maximum number of connections possible, N−1, for all nodes belonging to the network. This normalization allows us to compare networks with different sizes. Figure 2 shows the entropy-degree diagram for 3 networks that share the same number of nodes and links, have the same degree distribution but have different structures. Each marker (•) represents a node. Colors are used as a didactic artifact to improve the accessibility of visualization. They correspond to the maximum neighborhood radius ($r_{\max}$), that is, the greatest geodesic distance between the given node and the remaining nodes. The shaded regions follow the same colors and mark the space of possibilities for each value of $r_{\max}$. For example, the purple curve delimits the possible positions on the diagram for nodes with first and second neighbors. The region in blue indicates the positions for nodes with first, second and third neighbors and it follows for the others regions. The upper bounds are peaked at ($k\approx 1/r_{\max}$, $s\approx \ln r_{\max}$). Note the values do not depend on the network size; rather they depend only on the network structure. The magnitude of the increment in the geodesic entropy due to the increase of $r_{max}$ is inversely proportional to $r_{\max}$, ($\Delta s\approx r_{\max}^{-1}\Delta r_{\max})$. This means that there is a limit in which the increase of maximum geodesic distances (increase the sparsity or large world behavior) contributes significantly to the network entropy. The lower limits will be affected by the size of the network and converge to the first curve ($r_{\max}=2$) for large networks. See Figure 3. The entropy-degree diagram is a bidimensional representation of the statistics of distances and connectivity of all nodes. Both local and global properties of the entire network are visually represented. Higher geodesic entropy reflects a greater diversity of influences regarding the more homogeneous distribution in the population density across the neighborhood radius.The influence of nodes with high entropy into the networks are more diverse, perhaps more intricate and with greater variability. They are embedded in the structure in a site where the diversity of influences is more smoothly distributed. The overall relation of this node with the remain nodes is less trivial to predict. The opposite can be affirmed to nodes with low entropy.

## 3. Results

### Geodesic Entropy of Functional Brain Networks under Ayahuasca Influence

Next, we use the geodesic entropy to evaluate functional brain networks of the same group subjects in two different states of consciousness: before and 40 minutes after the ingestion of the psychedelic brew Ayahuasca.

**Figure 4:**
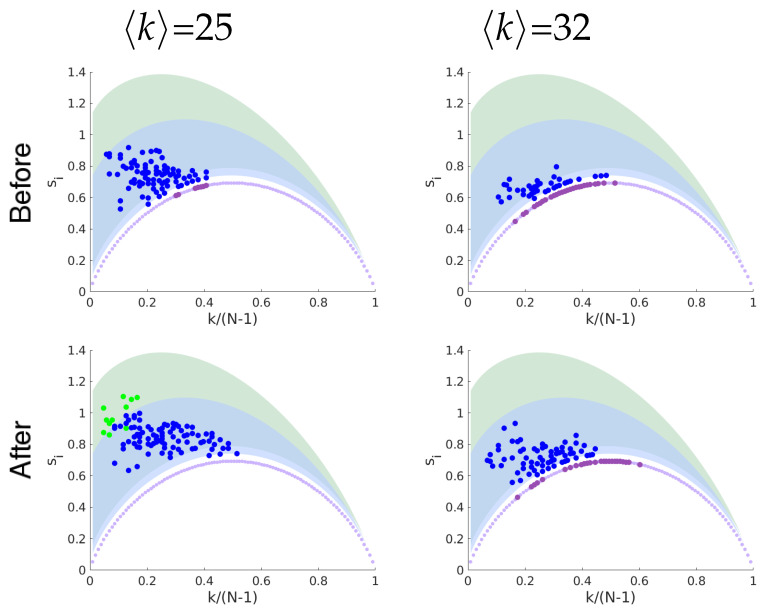
Entropy-degree diagram for a subject before (upper row) and after (bottom row) Ayahuasca intake for networks with mean degree 〈k〉=25 and 〈k〉=32 respectively. The colors follow the same rules of Figure 2. Note the nodes after Ayahuasca tends to populate regions in the diagram of higher entropy. The reader can find the diagrams for all subjects in the supplementary material.

**Figure 5:**
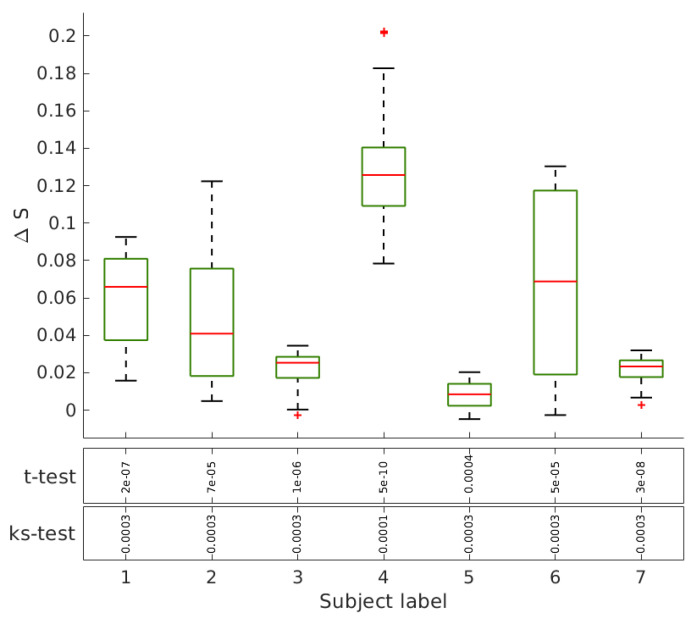
Characteristic geodesic entropy before and after Ayahuasca. The boxplot shows the averaged geodesic entropy divergence ($\Delta S=S_{\mathrm{after}}-S_{\mathrm{before}}$) considering 16 networks with the mean degree from 〈k〉=24 to 〈k〉=39 for each subject. The median value increases for all of them.

**Figure 6:**
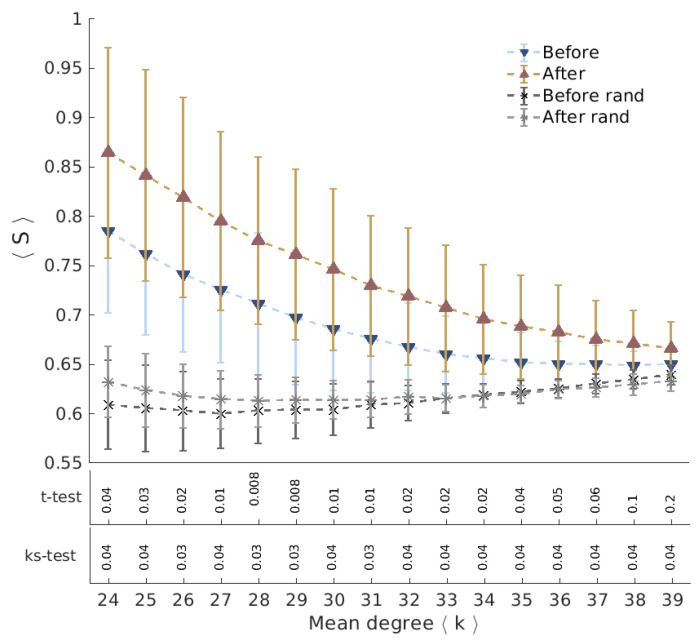
Average characteristic geodesic entropy before and after Ayahuasca. The graphic shows the average characteristic geodesic entropy under all subjects contrasting before and after Ayahuasca for networks with different densities (mean the degree from 〈k〉=24 to 〈k〉=39). The average characteristic geodesic entropy is greater for all network densities. The black and gray curves show the characteristic geodesic entropy for the degree-preserving random rewired versions of the networks before and after Ayahuasca respectively. The divergence between the randomized curves is not significant.

Figure 4 shows for illustration the entropy-degree diagram of one of the subjects before and after Ayahuasca intake for networks with mean degree 〈k〉=25 and 〈k〉=32. Note that the nodes in the entropy-degree diagram after Ayahuasca tend to have higher entropy. All subjects presented similar behavior. See supplementary material. Figure 5 shows the difference of the characteristic geodesic entropy after and before Ayahuasca for each subject by comparing pairs of networks with the same density of links. The boxplot depicts the distribution of characteristic geodesic entropy differences ($\Delta S=S_{\mathrm{after}}-S_{\mathrm{before}}$) of networks with the same mean degree. Note that the characteristic geodesic entropy increases for all subjects after Ayahuasca intake. Figure 6 shows the contrast of the characteristic geodesic entropy of networks with the same mean degree (same densities of links, tuned varying the threshold) averaged over all subjects before (blue) and after (brown) Ayahuasca intake. The increase suggests that characteristic geodesic entropy of functional networks under Ayahuasca influence tends to be higher than in ordinary condition.

The black and gray curves show the characteristic geodesic entropy for the randomized versions of the networks before and after Ayahuasca respectively. We use the Sneppen-Maslov algorithm [23] to randomize the links of networks keeping their degree distribution unchanged. In other words, the Sneppen-Maslov randomization breaks all structural features that do not depend on the degree distribution. Note that the randomization reduces the entropy in both conditions and no considerable divergence was found between the randomized curves. These results mean that the change in geodesic entropy we detected before and after Ayahuasca intake is related to underlying trends of the network structure. They do not result from the known changes in degree distribution [22].

## 4. Discussion and Conclusions

In a networked system, the role of each element depends on its relative position inside the overall network architecture. In such a connected system, the elements (represented by nodes) are influenced not only by first neighbors but may also be influenced indirectly by more distant nodes. The geodesic entropy is a statistical quantity that measures, in the frame of reference of a given node, the level of constraints in the (average of the) aggregated influences imposed by the distribution of neighborhood radius.

Functional networks are usually defined according to statistical dependencies between brain regions activities. Assumed as a fully connected network, all pairs of brain regions will have some interdependence. This can be measured directly, by means of the correlation between their activities, or inferred indirectly from the network structure. In this precise sense, the increase of the characteristic geodesic entropy in the functional networks after Ayahuasca intake indicates that the overall interdependencies are less constrained than before Ayahuasca ingestion.

We briefly compare and relate the geodesic entropy to similar quantities that have been used to study networks. The use of geodesic distances to evaluate Shannon entropy was proposed by Chen and collaborators [24]. Instead of defining the entropy per node, they defined a global entropy considering only one specific value *r* of geodesic distance. A recent work from Stella and Domenico proposes to calculate the Shannon entropy of distances to characterize centrality [25]. Their entropy is insensitive to the maximum neighborhood radius variability, due to normalization in the formula, by the factor log($r_{max}-1$). In contrast with the above methods, the geodesic entropy proposed in this work allows to evaluate the statistics of influences diversity for each node in a network accounting the full information of its geometry.

We have evaluated the geodesic entropy of functional brain network of subjects in the resting state before and after the ingestion of the psychedelic brew Ayahuasca. We have found that during Ayahuasca experience nodes of the functional network tend to have greater geodesic entropy than in the ordinary condition, resulting in networks with higher characteristic geodesic entropy. Hence, the geodesic distances between nodes become less constrained on average, i.e., their distribution becomes “wider”. In a previous work, we showed that the entropy of the degree distribution of brain functional connectivity networks under the influence of Ayahuasca is greater than in the ordinary state [22]. The entropy of degree distribution is a global measurement and networks with different patters can share the same degree distribution. The results presented here suggest that the structural patterns are less constrained under the influence of Ayahuasca compared to the ordinary condition. The geodesic distances are more broadly distributed, thus contributing to a high diversity of influences among the network nodes.

The hypothesis of an entropy increase in response to psychedelic states has been discussed in the literature [26,27,28]. This hypothesis predicts that the psychedelics state is associated with greater entropy compared to the ordinary state. The hypothesis of brain entropy increase claims to explain the increased flexibility in thoughts, facility to access suppressed memory, increase of creativity, among others [26].

In conclusion, we have shown how the geodesic entropy quantifies locally the overall influences of a network. Furthermore, we have used entropy-degree diagrams to evaluate features of each node in the network, giving a clearer view of the network topology and global connectivity. The application to fMRI-based functional connectivity networks sheds insights on how the brain changes under the influence of external influences. Although in this study we used Ayahuasca, there is in fact no reason why the method cannot be applied to study a variety of drugs, psychopathological symptoms, meditative states, etc. We hope that these ideas and methods find use in furthering our understanding of brain function networks and complex networks in general.

## 5. Materials and Methods

### 5.1. Data

The experimental procedures were performed in accordance with the guidelines and regulations approved by the Ethics and Research Committee of the University of São Paulo at Ribeirão Preto (process number 14672/2006). All volunteers signed a written informed consent. The fMRI data were acquired from 10 healthy adult volunteers (mean age 31.3, from 24 to 47 years, 5 women) with no history of neurological or psychiatric disorders—evaluated by DSM-IV structured interview [29]. They have at least 8 years of formal education and minimum Ayahuasca use time of 5 years. They were in absence of any medication for at least 3 months prior to the acquisition and also had not taken nicotine, caffeine, nor alcohol prior to the acquisition. Each volunteer ingested about 120–200 mL (2.2 mL/kg of body weight) of Ayahuasca. The chromatography analysis detected on the brew 0.8 mg/mL of DMT, 0.21 mg/mL of harmine and no harmaline at the threshold of 0.02 mg/mL [30]. The volunteers were submitted to two sections of fMRI scanning: one before and other 40 minutes after Ayahuasca intake when the subjective effects can be observed. In both cases, volunteers were requested to be in an awake resting state, that is, lying with their eyes closed, without performing any task. The samples of one volunteer were excluded from the dataset due to excessive head movement.

### 5.2. Obtaining Functional Networks from fMRI Data

The methods to extract the networks from the fMRI data used here are the same performed in reference [22]. The pre-processing of fMRI data was made according to standard guidelines. We performed spatial smoothing (Gaussian kernel, FWHM = 5 mm) and correction of slice-timing and head motion. We evaluated 9 regressors using a General Linear Model (GLM): 6 regressors to movement correction, 1 to white matter signal, 1 to cerebrospinal fluid and 1 to global signal (We used FSL Software, a free library of statistical tools available by Oxford Centre for Functional MRI of the Brain (http://www.ndcn.ox.ac.uk/divisions/fmrib)). The images were spatially normalized according to the Montreal Neurological Institute (MNI152 template) anatomical standard space using a linear transformation. We evaluated the band-pass filter using maximum overlap wavelet transform (MODWT), considering the Daubechies wavelet to split the signal into 4 scales of distinct frequency bands. We choose the scale 3 (frequency band ≈ 0.03–0.07 Hz) to be in agreement with the literature that considers the low frequency (≈0.01 to 0.1 Hz), preeminent on resting states [31].

We delimited 110 cortical anatomical regions according to the Harvard-Oxford cortical and subcortical structural atlas (threshold of >25%, using FMRIB software, an FSL library). We evaluated only 104 cortical regions because of an acquisition limitations for some subjects. The cortical regions were used to define the nodes of the brain networks and the correlation between their signals to define the links. The signals corresponding to each cortical region were obtained averaging the time series of all voxels (3D regular grid) into them (using Marsbar, SPM toolbox). We calculated the Pearson correlation of temporal series of all possible pairs of cortical regions, yielding a cross-correlation matrix. Thus, we have for each sample (before and after Ayahuasca) a 104 × 104 correlation matrix considered as an estimation of the brain functional connectivity. Since the cortical regions define the nodes, the correlation matrices were used to define the links of the functional brain networks.

For each sample, we generated a set of symmetric binary adjacency matrices by thresholding the absolute value of their correlation matrices. Precisely, whether the absolute value of the element matrix is larger than the defined threshold, a link is formed ($A_{i,j}=1$), otherwise, no link is formed ($A_{i,j}=0$). We choose a range of thresholds that ensure the networks were fully connected but also sparse. We adopted the same criteria as in references [22,32,33,34]. We summarized bellow the pipeline. Further details about the computational methods, randomized network model, sensitivity analysis, etc. can be found in the reference [22]. We considered the network with lower global efficiency and greater local efficiency than its degree-preserving random rewired version (Sneppen-Maslov algorithm) [23]. We fixed the same band of correlation thresholds for all samples, allowing a more accurate comparison. It was necessary to exclude two subjects from our analysis due to a trade-off in the threshold range, leaving 7 subjects for the analysis (4 women). Since we intended to evaluate the difference between topological features of networks before and after Ayahuasca intake, we compare networks with the same density of links. The chosen threshold correlation range is 0.28≤η≤0.37 that yield networks with mean degree in the range 24≤〈k〉≤39. Summarizing, we created two sets of networks (before and after Ayahuasca intake) that allow 16 different comparisons (i.e., of differing mean degrees) for each subject’s sample.

References

## Supplementary Materials

The following are available online at http://www.mdpi.com/1099-4300/21/2/128/s1.
