# Supplementary material for: Characterizing Complex Networks Using Entropy-Degree Diagrams: Unveiling Changes in Functional Brain Connectivity Induced by Ayahuasca
Source: Entropy (Basel). 2019 Jan 30;21(2):128. doi: 10.3390/e21020128 (PMC7514607; doi:10.3390/e21020128)
Supplement: Supplementary file 1 [file entropy-21-00128-s001.pdf]

Supplementary Materials: Characterizing complex networks using entropy-degree diagrams: unveiling changes in functional brain connectivity induced by Ayahuasca

A. Viol [1,2*],Fernanda Palhano-Fontes [3], Heloisa Onias [3], Draulio B. de Araujo [3] Philipp Hövel [1,2,4] and G. M. Viswanathan [5,6]

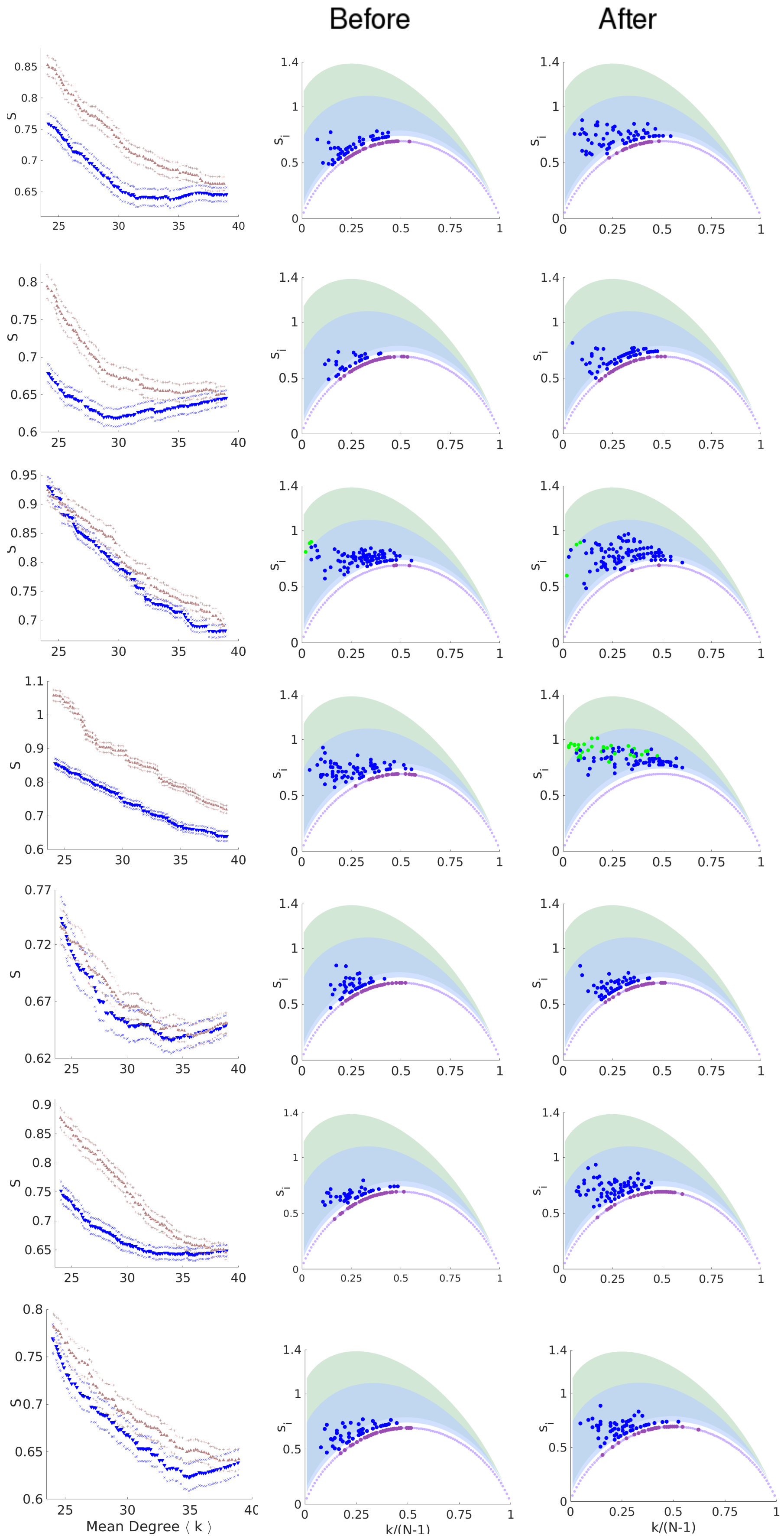

Figure 7. See next page for caption.

Figure S1. Geodesic entropy before and after Ayahuasca intake for all 7 subjects. The first column depicts the curves to characteristic geodesic entropy for networks with the mean degree from $\langle k \rangle = 24$ to $\langle k \rangle = 39$ for before (blue) and after (brown) Ayahuasca intake. The second and third columns show the entropy-degree diagram for before and after respectively for networks with mean degree $\langle k \rangle = 32$. Note an increase of geodesic entropy for all subjects.
